# Survival Outcomes of Patients With Stage IB3 Cervical Cancer Who Undergo Abdominal Radical Hysterectomy Versus Radiochemotherapy

**DOI:** 10.3389/fonc.2022.933755

**Published:** 2022-07-06

**Authors:** Zhiqiang Li, Qing Yang, Jianxin Guo, Guoqiang Liang, Hui Duan, Shaoguang Wang, Min Hao, Wentong Liang, Donglin Li, Xuemei Zhan, Qinghuang Xie, Jinghe Lang, Ping Liu, Chunlin Chen

**Affiliations:** ^1^ Department of Obstetrics and Gynecology, Nanfang Hospital, Southern Medical University, Guangzhou, China; ^2^ Department of Obstetrics and Gynecology, Shengjing Hospital of China Medical University, Shenyang, China; ^3^ Department of Obstetrics and Gynecology, Daping Hospital, Army Medical University, Chongqing, China; ^4^ Department of Gynecology, Yantai Yuhuangding Hospital, Yantai, China; ^5^ Department of Gynecology, the Second Hospital of Shanxi Medical University, Taiyuan, China; ^6^ Department of Obstetrics and Gynecology, Guizhou Provincial People’s Hospital, Guizhou, China; ^7^ Department of Gynecology, Jiangmen Central Hospital, Jiangmen, China; ^8^ Department of Gynecology, Foshan Women and Children Healthcare Hospital, Foshan, China; ^9^ Department of Obstetrics and Gynecology, Peking Union Medical College Hospital, Peking Union Medical College, Beijing, China

**Keywords:** cervical cancer, abdominal radical hysterectomy, radiochemotherapy, overall survival, disease-free survival, stage IB3 cervical cancer

## Abstract

**Objective:**

This study aimed to compare the survival outcomes among stage IB3 cervical cancer patients who undergo abdominal radical hysterectomy (ARH)+pelvic lymphadenectomy ± para-aortic lymph node dissection versus radiochemotherapy (R-CT).

**Methods:**

Based on the large number of diagnoses and treatments for cervical cancer in the Chinese database, propensity score matching (PSM) was used to compare the 5-year overall survival (OS) and disease-free survival (DFS) rates of the ARH group and R-CT group.

**Results:**

There were 590 patients with stage IB3 cervical cancer according to the FIGO 2018 staging system, with 470 patients in the ARH group and 120 patients in the R-CT group. The ARH and R-CT groups showed different 5-year OS and DFS rates in the total study population, and the 5-year OS and DFS rates in the R-CT group (n = 120) were lower than those in the ARH group (n = 470) (OS: 78.1% vs. 92.1%, p < 0.001; DFS: 71.6% vs. 90.3%, p < 0.001). R-CT was associated with a worse 5-year OS rate (hazard ratio [HR] = 3.401; 95% confidence interval [CI] = 1.875–6.167; p < 0.001) and DFS rate (HR = 3.440; 95% CI = 2.075–5.703; p < 0.001) by Cox multivariate analysis. After 1:3 PSM, the 5-year OS and DFS rates in the R-CT group (n = 108) were lower than those in the RH group (n = 280) (OS: 76.4% vs. 94.0%, p < 0.001; DFS: 69.3% vs. 92.6%, p < 0.001, respectively). R-CT was associated with a worse 5-year OS rate (HR = 4.071; 95% CI = 2.042–8.117; p < 0.001) and DFS rate (HR = 4.450; 95% CI = 2.441–8.113; p < 0.001) by Cox multivariate analysis.

**Conclusion:**

Our study found that for FIGO 2018 stage IB3 cervical cancer patients, ARH resulted in better OS and DFS than R-CT.

## Introduction

Cervical cancer is a common malignant tumor of the female genital tract and the fourth leading cause of cancer death among women worldwide, especially in developing countries (1). In 2018, FIGO updated their clinical classification system. The following main changes were incorporated: the use of any imaging modality and/or pathological findings for judging the stage. For stage IB disease, the width of the lesion is no longer taken into consideration. Stage IB now includes three subgroups based on tumor size increases of 2 cm: stage IB1 (≤2 cm), stage IB2 (>2 to ≤4 cm) and stage IB3 (>4 cm). The most relevant modification was the introduction of the lymph node (LN) status; indeed, LN involvement (*via* histological or radiological assessment) was specifically designated as stage IIIC disease (IIIC1 pelvic LN metastasis and IIIC2 para-aortic LN metastasis) (1, 2). As a result, a new problem has arisen—that is, whether stage IB2 treatment recommendations based on the old staging system are still suitable based on the new staging system. Based on the clinical diagnoses and treatments for cervical cancer in the Chinese (Four C) database, this paper compared ARH versus R-CT for stage IB3 cervical cancer patients based on the new FIGO 2018 staging system and explored appropriate treatment strategies for this patient population.

## Materials and Methods

### Data Source

The establishment of the cervical cancer database was reviewed by the Ethics Committee of Nanfang Hospital, Southern Medical University (ethics number NFEC-2017-135), and written informed consent was waived by the ethics committee. The clinical trial identifier is CHiCTR1800017778 (International Clinical Trials Registry Platform Search Port, http://apps.who.int/trialsearch/). For the data collection methods and database establishment methods, please refer to the previously published articles by our team (3–7). General patient clinical data, surgery-related data, pathological information, and 315 other parameters were used for the standardized training of gynecologists and by the participating units after training for prognostic follow-up. Follow-up was mainly carried out *via* outpatient and telephone follow-up, and survival, recurrence, and other information were recorded. From 2004 to 2018, 63,926 cases of cervical cancer were collected across 47 hospitals in China.

### Inclusion and Exclusion Criteria

In this study, the inclusion and exclusion criteria were as follows.

ARH with postoperative standard therapy group (ARH group) (1): aged ≥ 18 years old (2); FIGO (2018) stage IB3 (3); histological type of squamous cell carcinoma, adenocarcinoma, or adenosquamous carcinoma (4); primary treatment with open surgery (5); no use of neoadjuvant therapy (6); QM-B or QM-C hysterectomy + pelvic lymphadenectomy ± para-aortic lymph node resection; and (7) postoperative standard adjuvant treatment according to the pathological factors described by the guidelines.

R-CT group (1): aged ≥ 18 years old (2); FIGO (2018) stage IB3 (3); histological type of squamous cell carcinoma, adenocarcinoma, or adenosquamous carcinoma (4); primary treatment with R-CT; and (5) a radiotherapy dose ≥ 45 Gy.

The exclusion criteria were as follows (1): patients who did not meet the above inclusion criteria and (2) pregnant patients with cervical cancer, and patients with the accidental discovery of cervical cancer, stump cancer, or other types of malignant tumors.

### Observation Indicators

The observation endpoints were overall survival (OS) and disease-free survival (DFS), and the cutoff point for long-term oncological outcomes was 5 years. OS was defined as the date of diagnosis until death from any cause or the last effective follow-up, and DFS was defined as the date of diagnosis until death, recurrence, or the last effective follow-up.

### Statistical Methods

SPSS software (Version 22.0, SPSS Inc., Chicago, IL, USA) was used for statistical analysis, and the PSM extension of SPSS 22.0 software was used to perform propensity score matching (PSM). Measurement data are expressed as the mean ± standard deviation, and an independent sample t test was used for comparisons between groups. Count data are expressed as percentages (%), and the chi-square test was used to compare intergroup rates. Kaplan–Meier curves were drawn to analyze survival, and log-rank tests were used to compare differences in the survival curves. Multivariate Cox regression was used to analyze and determine the independent risk factors, relevant risks, and confidence intervals. In this study, p < 0.05 was considered statistically significant.

## Results

### Case Screening Results

**Figure 1:**
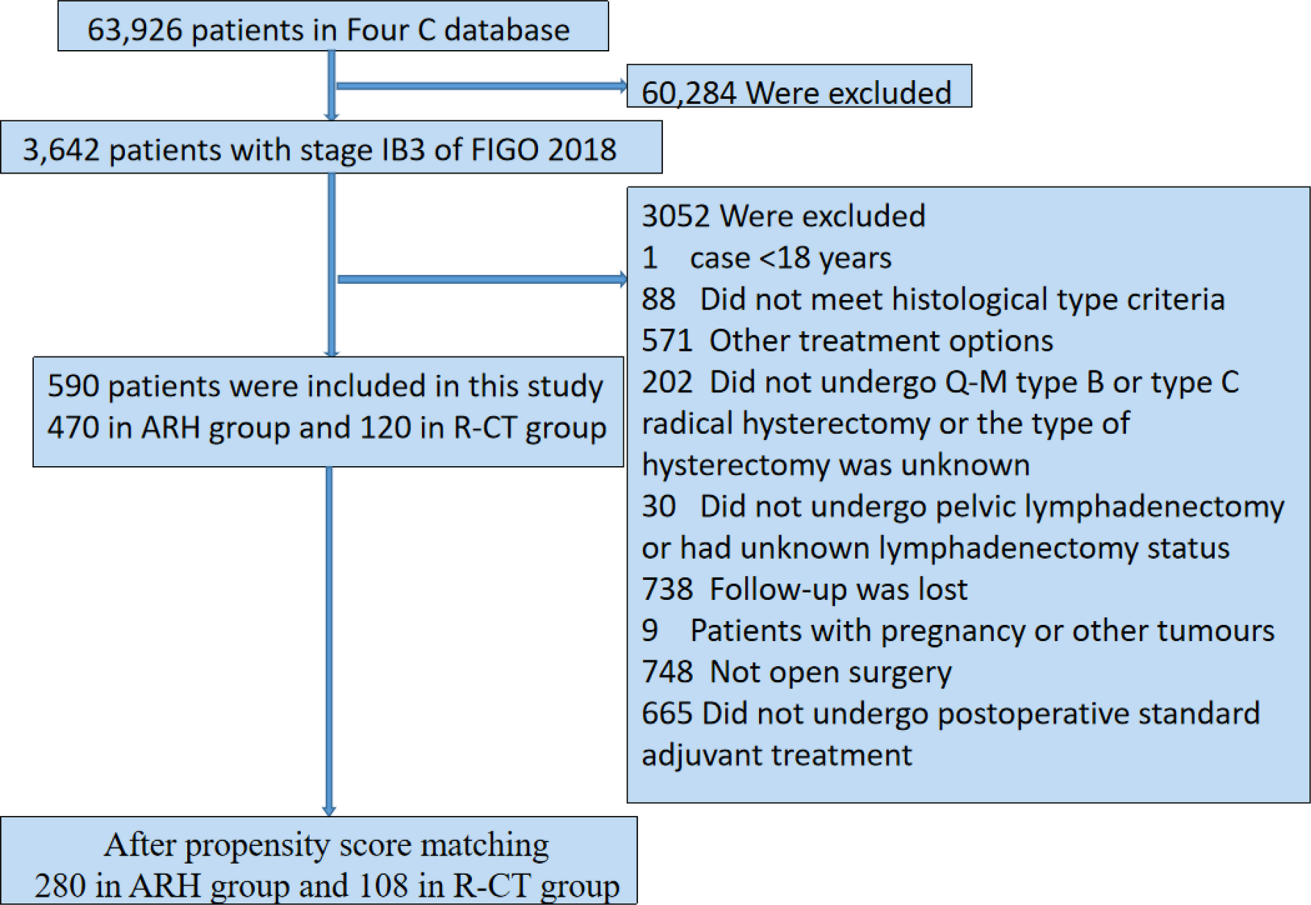
Flow diagram of recruitment and exclusions. ARH, abdominal radical hysterectomy; R-CT, radio-chemotherapy.

A total of 590 patients met the inclusion and exclusion criteria (470 in the RH group and 120 in the R-CT group) **(** **Figure 1** **)**.

### Oncological Outcome Comparison of the ARH Group and the R-CT Group Before and After Matching

**Figure 2:**
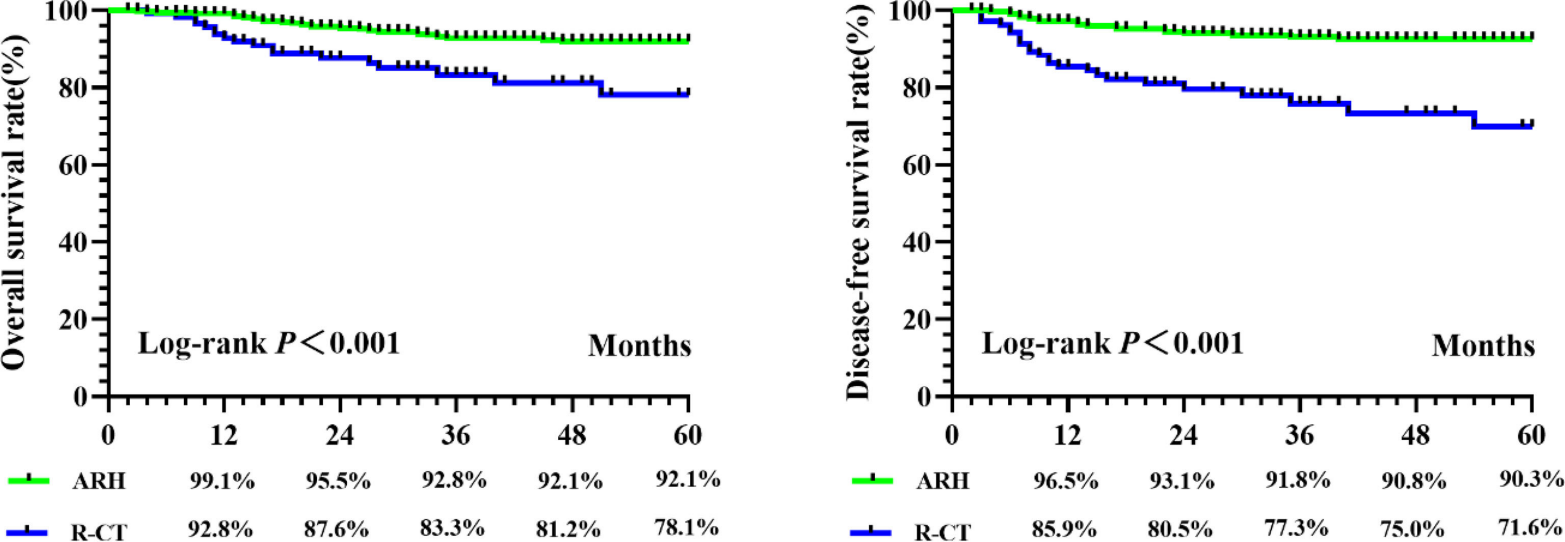
The 5-year OS and DFS in ARH group and R-CT group before PSM.

**Figure 3:**
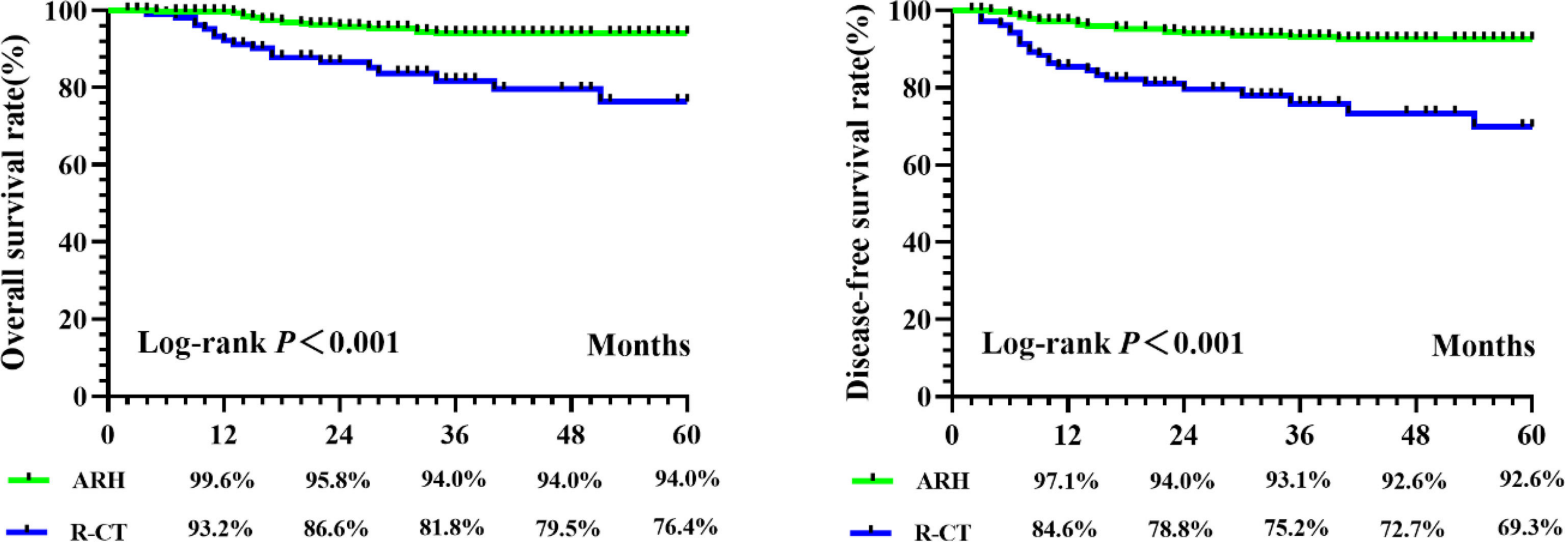
The 5-year OS and DFS in ARH group and R-CT group after PSM.

A total of 590 patients met the entry criteria: 470 were included in the ARH group, and 120 were included in the R-CT group. Baseline analysis showed that there were differences in the baseline parameter of age between the two groups **(** **Table 1** **)**. Patients in the ARH group (47.11 ± 8.294 years) were younger than those in the R-CT group (50.54 ± 10.855 years) (p < 0.001). The baseline distribution of histological type and age was not balanced among the 590 patients who were included. To reduce the influence of confounding factors, we performed 1:3 PSM and then performed a survival analysis. After 1:3 PSM, 280 patients were included in the ARH group, and 108 patients were included in the R-CT group. The baseline analysis between the two groups was not statistically significant (p > 0.05) **(** **Table 1** **)**. Among the total study population, the difference in survival outcomes was statistically significant between the ARH group (n = 470) and the R-CT group (n = 120) (OS 92.1% vs. 78.1%, p < 0.001; DFS 90.3% vs. 71.6%, *p* < 0.001) (**Figure 2**). Cox multivariate analysis indicated that the risk of death in the R-CT group was higher than that in the ARH group; for the R-CT group, the 5-year OS and DFS outcomes were independent risk factors (OS: HR = 3.401; 95% CI, 1.875–6.167; p = 0.001; HR = 3.440; 95% CI, 2.075–5.703, p < 0.001) **(** **Table 2** **)**. After 1:3 PSM, survival analysis showed that the 5-year OS and 5-year DFS in the ARH group were higher than those in the R-CT group (OS: 94.0% vs. 76.4%, p < 0.001; DFS: 92.6% vs. 69.3%, p < 0.001) **(** **Figure 3** **)**. Cox multivariate analysis indicated that for the R-CT group, the 5-year OS and DFS outcomes were independent risk factors (OS: HR = 4.071, 95% CI: 2.042–8.117, p < 0.001; DFS: HR = 4.450, 95% CI: 2.441–8.113, p < 0.001) **(** **Table 3** **)**.

**Table 1:** Data of the ARH group and R-CT group patients before and after matching.

Variables	Unmatched			Matched		
	ARH (n = 470)	R-CT (n = 120)	p-value	ARH (n = 280)	R-CT (n = 108)	p-value
Age (years)	47.11 ± 8.294	50.54 ± 10.855	<0.001	48.10 ± 8.003	48.59 ± 8.715	0.410
Histological type			0.142			0.838
Squamous cell carcinoma	411 (87.5%)	112 (93.4%)		258 (92.2%)	101 (93.5%)	
Adenocarcinoma	42 (8.9%)	7 (5.8%)		20 (7.1%)	6 (5.6%)	
adenosquamous carcinoma	17 (3.6%)	1 (0.8%)		2 (0.7%)	1 (0.9%)	

**Table 2:** COX Multivariate analysis of the overall study population according to group.

Variables	5-year OS				5-year OS			
Before matching	p	HR	95% CI%		p	HR	95% CI	
**ARH group vs. R-CT group**	<0.001	3.401	1.875	6.167	<0.001	3.710	2.219	6.204
Age	0.030	0.965	0.934	0.996	0.041	0.971	0.944	0.999
Histological type								
Squamous cell carcinoma	0.030				0.058			
Adenocarcinoma	0.948	1.040	0.320	3.385	0.310	1.554	0.663	3.641
Adenosquamous carcinoma	0.008	4.090	1.439	11.626	0.025	3.244	1.160	9.070

**Table 3:** COX multifactor analysis of matched patients.

Variables	5-year OS				5-yearDFS			
After matching	p-value	HR	95% CI		p-value	HR	95% CI	
**ARH group vs. R-CT group**	<0.001	4.071	2.042	8.117	<0.001	4.450	2.441	8.113
Age	0.005	0.940	0.900	0.982	0.035	0.951	0.925	0.997
Histological type								
Squamous cell carcinoma	0.090				0.159			
Adenocarcinoma	0.514	1.623	0.379	6.950	0.185	2.025	0.713	5.754
Adenosquamous carcinoma	0.032	9.317	1.205	72.049	0.140	4.541	0.609	23.876

## Discussion

The NCCN guidelines for stage IB3 cervical cancer recommend the first-choice treatment of concurrent radiotherapy and chemotherapy (evidence level 1) and the secondary-choice treatment of extensive hysterectomy PL ± PAL (evidence level 2B) (8, 9). Some controversy remains regarding neoadjuvant chemotherapy, and conflicting findings have been reported. The NCCN guidelines do not recommend neoadjuvant chemotherapy for cervical cancer (10) The FIGO guidelines also recommend that neoadjuvant chemotherapy be used only in areas lacking radiotherapy equipment and in clinical trials. Our study shows that patients with stage IB3 cervical cancer (FIGO 2018) treated with radical hysterectomy have good survival outcomes. Based on a sufficiently large sample size, long-term effective follow-up, and strict control of bias through tendency score matching in the analysis process, the analysis of this study has high credibility.

Although there are no studies discussing the survival outcomes of ARH versus R-CT in patients diagnosed based on the new 2018 FIGO stage IB3 classification system, previous research on patients diagnosed based on the 2009 FIGO stage IB2 classification system also has reference value. Stage IB2 (>4 cm) in the original staging system was changed to stage IB3 (>4 cm) in the 2018 system and did not include lymph node metastasis (11). Previous studies have shown that the 5-year OS rate of patients classified as having FIGO 2009 stage IB2 disease after ARH was 72% to 72.8% (12). The Melissa Bradbury study found that the OS rate of women with stage IB2 disease who underwent ARH during the 2009 FIGO staging period was higher than that of women who underwent R-CT (74.6%:60.0%, p = 0.05), which is consistent with the results of this study to some extent (13). The 5-year OS of patients with stage IB3 disease in this study was higher than that of patients with stage IB2 based on the previous (2009) staging system, which may explain the elimination of lymph node metastasis in the new staging system (14).

Considering time and economic costs, direct radical hysterectomy is preferred for FIGO 2018 stage IB3 disease, offering a new direction for the treatment of this patient population. Rocconi’s cost–benefit analysis of the treatment of 2009 FIGO stage IB2 cervical cancer showed that, compared with primary radiotherapy or neoadjuvant chemotherapy, early radical hysterectomy is the most cost-effective strategy, followed by radical hysterectomy and adjuvant radiotherapy and chemotherapy (15). The current study also provides evidence to support this finding.

Compared with previous research reports and articles on the treatment of cervical cancer, this study has some advantages, but there is still a lack of international literature on treatment strategies for patients with FIGO 2018 stage IB3 disease. First, this is one of a few large, international real-world cervical cancer studies, contributing to a more complete clinical diagnosis and treatment database for cervical cancer. Second, due to the sufficient number of included patients, cervical cancer at each stage could be analyzed from many angles, levels, and directions. Third, we used the PSM method to balance baseline differences on the basis of real-world research methods, making the results more accurate. Fourth, only open-surgery cases were included because the LACC study found that laparoscopic surgery had worse oncological outcomes than open surgery (16), for possible reasons including tumor spillage, CO_2_ circulation of tumor cells, and other factors (17). Our study was one of the first population-based studies to compare the 5-year OS and DFS rates between ARH and R-CT in stage IB3 cervical cancer patients. A strength of the present study was its large sample size. Our study analyzed a large cohort of cervical cancer patients across 47 hospitals over a 14-year period. This study may be the first to discuss the survival outcomes of FIGO 2018 stage IB3 cervical cancer patients who undergo ARH and R-CT.

However, our research inevitably has some limitations. First, this is a retrospective study that may have confounding factors and bias; for example, patients in the R-CT group were older than those in the ARH group. However, we tried to balance these differences by PSM. Second, although this study comprised 590 hospitalized patients with cervical cancer in China, it did not fully cover all regions of China; however, the database is still representative of the diagnoses and treatments of cervical cancer patients in China. Third, this study did not take into account preoperative cervical conization, which has been found to affect oncological outcomes (18).

In summary, the 5-year OS and DFS rates of patients with stage IB3 cervical cancer, according to the FIGO 2018 classification system, who underwent ARH were superior to those of patients who underwent R-CT, indicating that ARH may offer better oncologic outcomes to patients with cervical cancer. This finding is different from the radiotherapy recommendations described in the NCCN guidelines. More prospective clinical studies are needed to confirm the optimal treatment strategy for patients with FIGO 2018 stage IB3 cervical cancer.

## Data Availability Statement

The original contributions presented in the study are included in the article/supplementary material. Further inquiries can be directed to the corresponding authors.

## Ethics Statement

This study was approved by the Ethics Committee of Nanfang Hospital affiliated with Southern Medical University (Guangzhou, China) (ethical number: NFEC-2017-135). Written informed consent for participation was not required for this study in accordance with the national legislation and the institutional requirements.

## Author Contributions

(I) Conception and design: CC, PL, JL; (II) administrative support: CC, PL; (III) provision of study materials or patients: XZ, DL, WL, MH, SW, HD, JL, PL, CC; (IV) collection and assembly of data: GL, JG, QY, ZL; (V) data analysis and interpretation: GL, ZL, QY, JG; (VI) manuscript writing: all authors. All authors contributed to the article and approved the submitted version.

## Funding

This study received funding from the National Science and Technology Support Program of China (2014BAI05B03), the National Natural Science Fund of Guangdong (2015A030311024), and the Science and Technology Plan of Guangzhou (158100075).

## Conflict of Interest

The authors declare that the research was conducted in the absence of any commercial or financial relationships that could be construed as a potential conflict of interest.

## Publisher’s Note

All claims expressed in this article are solely those of the authors and do not necessarily represent those of their affiliated organizations, or those of the publisher, the editors and the reviewers. Any product that may be evaluated in this article, or claim that may be made by its manufacturer, is not guaranteed or endorsed by the publisher.
